# Efficient automatic 3D segmentation of cell nuclei for high-content screening

**DOI:** 10.1186/s12859-022-04737-4

**Published:** 2022-05-31

**Authors:** Mariusz Marzec, Adam Piórkowski, Arkadiusz Gertych

**Affiliations:** 1 Faculty of Science and Technology, Institute of Biomedical Engineering, University of Silesia, Bedzinska St. 39, 41-200 Sosnowiec, Poland; 2 Department of Biocybernetics and Biomedical Engineering, AGH University of Science and Technology, Mickiewicza 30, 30-059 Cracow, Poland; 3 Department of Surgery, Department of Pathology and Laboratory Medicine, Cedars-Sinai Medical Center, Los Angeles, CA 90048 USA; 4 Faculty of Biomedical Engineering, Silesian University of Technology, Roosvelta 40, 41-800 Zabrze, Poland

**Keywords:** Image processing, Image analysis, 3D nuclei segmentation, Automated analysis, Bio-image informatics, High-content screening

## Abstract

**Background:**

High-content screening (HCS) is a pre-clinical approach for the assessment of drug efficacy. On modern platforms, it involves fluorescent image capture using three-dimensional (3D) scanning microscopy. Segmentation of cell nuclei in 3D images is an essential prerequisite to quantify captured fluorescence in cells for screening. However, this segmentation is challenging due to variabilities in cell confluency, drug-induced alterations in cell morphology, and gradual degradation of fluorescence with the depth of scanning. Despite advances in algorithms for segmenting nuclei for HCS, robust 3D methods that are insensitive to these conditions are still lacking.

**Results:**

We have developed an algorithm which first generates a 3D nuclear mask in the original images. Next, an iterative 3D marker-controlled watershed segmentation is applied to downsized images to segment adjacent nuclei under the mask. In the last step, borders of segmented nuclei are adjusted in the original images based on local nucleus and background intensities. The method was developed using a set of 10 3D images. Extensive tests on a separate set of 27 3D images containing 2,367 nuclei demonstrated that our method, in comparison with 6 reference methods, achieved the highest precision (PR = 0.97), recall (RE = 0.88) and F1-score (F1 = 0.93) of nuclei detection. The Jaccard index (JI = 0.83), which reflects the accuracy of nuclei delineation, was similar to that yielded by all reference approaches. Our method was on average more than twice as fast as the reference method that produced the best results. Additional tests carried out on three stacked 3D images comprising heterogenous nuclei yielded average PR = 0.96, RE = 0.84, F1 = 0.89, and JI = 0.80.

**Conclusions:**

The high-performance metrics yielded by the proposed approach suggest that it can be used to reliably delineate nuclei in 3D images of monolayered and stacked cells exposed to cytotoxic drugs.

## Background

High-content screening (HCS) platforms are currently state-of the art approaches for drug discovery and drug efficacy assessment. Examining the drug response on HCS platforms necessitates the quantification of fluorescent signals captured by automated microscopy systems in single cells. To enable quantification of the fluorescence in whole cells without losing the spatial information, modern screening platforms employ high-resolution, three-dimensional (3D) image capture. Segmentation of cell nuclei in 3D images is an essential prerequisite when quantifying the captured fluorescence in whole cells or sub-cellular compartments. However, this segmentation is challenging due to variabilities in cell confluency and morphological characteristics induced by chemotherapeutics, which are often highly cytotoxic. Another challenge that is commonly seen in acquired images is the gradual loss of fluorescence intensity and contrast with the depth of scanning. Despite advances in algorithms for segmenting nuclei in HCS, robust 3D methods that are insensitive to changes in size, shape, texture and staining intensity of nuclei induced by harsh experimental conditions are still lacking. Most of the existing methods can cope with some but not all of these issues. Others are slow and computationally expensive and therefore have limited applicability to HCS tasks.

3D HCS screening is a powerful tool for measuring drug response and assessment of cytotoxicity and cell viability [1–5]. Modern HCS platforms deliver 3D image data in a multiplex format with stacks of optical sections (z-stacks) organized into channels—one for each fluorophore. Based on the nuclear masks segmented from a 3D image, HCS can count and perform morphological and functional profiling of cells. A relatively large number of software platforms are available to generate measurements for HCS studies [6–10]. However, the problem is that different methods yield different count values, and the quality of delineations of 3D masks of nuclei vary when tested in images supplemented with ground truth.

To achieve reliable segmentation of nuclei, numerous semi-automated segmentation techniques have been developed [7, 8, 11–13]. However, many of them are tailored to a specific screening task or require prior setting of multiple parameters [3, 14]. Many fully automated methods are frequently restricted by the morphological characteristics of specimens and are thus able to analyse cells with less-complex patterns. Unlike in high-magnification images, where fine chromatin details can be quantified, nuclei in low-magnification images appear round and often uniformly intense without any visible chromatin texture. Yet, as image magnification increases, so does the level of visual detail in cells, which makes nuclei segmentation more challenging and prone to errors.

Existing 3D nuclei segmentation techniques can be divided into three main families: watershed-based, deformable model-based, and level-set-based segmentations [15–18]. Watershed-based approaches involve markers (seeds) and are computationally efficient. However, pipelines which use watershed-based segmentations frequently require pre- and post-processing procedures to deal with possible over- and under-segmentations [19–21]. The performance of watershed-based methods is often determined by the performance of the seed-generation technique that is run prior to the actual segmentation. For instance, one such seed-generation method was proposed in [11]: shape priors derived directly from a non-segmented image were funnelled as features ahead of the actual nuclear segmentation routines. In [11], the output of a 3D radial symmetry transform was used to approximate the location and shape of nuclei. Watershed-based pipelines can be improved by reducing the number of processing steps and hyperparameters that govern the segmentation [7, 8]. In [16], the authors presented an algorithm for multi-cell segmentation and tracking. This method was based on the coupled active surfaces framework. Connected objects determined in the first image frame are segmented with a level-set function. One level-set is assigned to each object. Each level-set function is iteratively evolved until convergence criteria are satisfied. It the next step, watersheds are used to perform rough splitting of level-set functions in connected components. The algorithm determines whether existing level-set functions need to be terminated or new functions should be introduced. In the final step, the Radon transform is used to separate the level-set functions of closely positioned nuclei.

Approaches that are based on deformable models require less pre- and/or post-processing but are more computationally intense, especially for 3D applications. Level-set-based segmentations utilize geometric active contours [22–24]. To track shapes, they connect pixels that have similar intensity, and they yield a 3D surface or a 2D contour that delineates individual nuclei. Except for a coupling constraint that inhibits overlapping of neighbouring contours, level-set methods do not require explicit parameterization and can yield masks for objects with complex shapes. However, they are computationally expensive because each object (nucleus) has to be represented by a unique level-set function. These requirements increase the computational burden and thus often prohibit the use in HCS applications of level-set methods for specimens with high cell confluency. In [25], the authors proposed a software system named FARSIGHT, which was used for segmentation of various types of three-dimensional (3D) bioimages. It utilized the graph cuts method to separate objects from the background, and a clustering algorithm was used to generate a mask of seeds employed in the seeded watershed segmentation.

Another method [26] has been used to separate clustered nuclei from fluorescence microscopy cellular images. The authors proposed shape markers and a marking function in a watershed-like algorithm. Geometric active contours were used to initially segment 2D images; then, an adaptive H-minima-based algorithm was used to find shape markers that served as seeds for watershed-based splitting of closely spaced nuclei. This method was adapted to process 3D images.

Deep-leaning (DL) based methods for 3D segmentation of cell nuclei are the most recent. They utilize artificial neural network models with convolutional and feature extraction layers that perform semantic segmentation of the volumetric image data on the network input and yield a 3D mask of nuclei on its output. Their main advantage is the automatic extraction of learnable image features that the model automatically distils based on the training data in order to distinguish the nucleus from background and separate one nucleus from another in aggregates [27–30]. These approaches utilize variants of the 3D-UNet [31] or Fully Convolutional Neural Network (FCNN) architectures [32] in the model’s backbone. Although the DL approaches for 3D nuclei segmentation perform well on images of highly confluent cells, they achieve higher segmentation accuracy when the nuclear mask or probability scores of the detected nuclei at the model output is converted to a set of markers (seeds) for use with watershed segmentation which completes the segmentation. Examples of this two-step approach can be found in [27, 28, 30]. To learn robust features, the 3D DL models need a significant amount of training data, that is images with every nucleus delineated in 3D. However, this process is time consuming and costly because delineations are often generated manually.

We have developed a new algorithm to robustly delineate and separate nuclei in 3D images of cellular specimens. The algorithm was tested on image data from experiments that tested DNA demethylating drug screening on human cells. It is based on voxel intensity thresholding to generate a preliminary nuclear mask; this is followed by iterative 3D marker-controlled watershed segmentation to separate the nuclei of adjacent cells. In the last step, borders of segmented nuclei are adjusted based on local nucleus and background intensities. This approach makes it robust against local changes in image contrast and intensity. This method is generally dedicated to segmentation of cell nuclei in experiments with stacked and closely adjacent cells grown on plates in wells that are stained and subsequently imaged in 3D. In comparison to the existing methods, the proposed approach requires only a small set of shape priors. Our approach was developed and tested on high-resolution, 3D, confocal images of human-derived cells that had been exposed to anticancer drugs. The nuclei delineation performance of the proposed method was compared to state-of-the-art methods, including one previously developed by the authors [11]. Our method was also compared with two DL techniques. We chose QCANet [27] and 3DCellTracker [28] because their software frameworks were made publicly available. In order to apply these methods, our image data were respectively adjusted. To compare results of segmentations by these methods, our images were downsized (to achieve similar sizes of voxels of the image data used in training) and normalized before segmentation. In case of referenced methods, the segmentation was carried out with default set of parameters for each method.

## Results

**Fig. 1:**
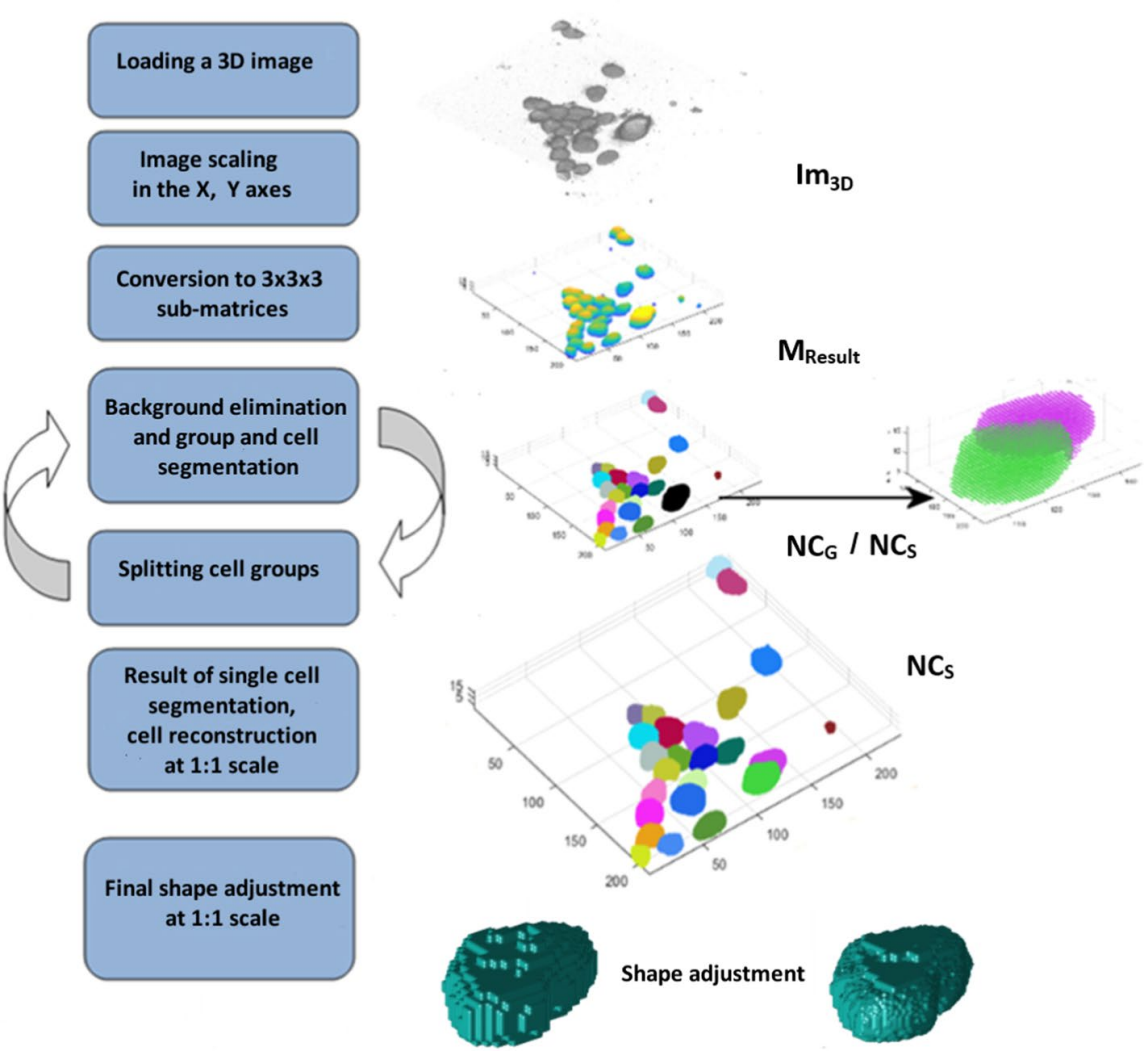
Block diagram of the cell nuclei segmentation algorithm and its individual stages

The aim of the research was to develop a fast and effective nuclei segmentation method for 3D cell specimens. The proposed segmentation method is divided into three main stages. First, the algorithm analyses the entire 3D image matrix, $Im_{3D}$, in order to determine the statistical parameters of the image and eliminate the background. This stage is performed on a downsampled image. The second stage involves segmentation, which leads to accurate separation of individual nuclei. Finally, the algorithm reconstructs the cells and then upsamples the obtained 3D masks to the original image resolution. Figure 1 shows a detailed block diagram of the developed segmentation algorithm. Initially, the analysed 3D image, $Im_{3D}$, is reduced by 50% in the *XY* axes; the *Z* axis remains unchanged. As a result, the memory requirements and the time needed for image analysis are reduced by up to 4 times. For the reduced matrix of the 3D image (after scaling, $Im_{3Dsc}$), the maximum intensity of all image voxels, $I_{Max}$, is determined. The 3D image matrix, $Im_{3Dsc}$, is then converted to a set of sub-matrices that have a size of $3{\times }3{\times }3$ voxels. Average intensity is determined for each of these sub-matrices. Next, based on an automatically determined initial global threshold, they are classified as the background or nucleus. This value is calculated by combining the results of two selected global thresholding methods (see the “Methods” section for details). The thus-classified sub-matrices containing nuclear voxels are recorded in the resulting matrix, $M_{Result}$, and are analysed for single nuclei or group membership. The groups of nuclei are then divided (into single nuclei) and the precise shapes of single segmented nuclei are determined. Each group of voxels is analysed for shape and size and is classified as either a single nucleus ($NC_{S}$) or a group of nuclei ($NC_{G}$). All $NC_{G}$ voxels are re-segmented with the same algorithm but with a higher threshold until further splitting of the cell group is impossible and the nuclei are classified as $NC_{S}$.

The end result of the algorithm’s operation is a 3D mask of nuclei, $NC_{S}$, in the examined 3D image. The separated nuclei are marked with indexes, each containing information about the complete voxel list and nuclear size. The last stage involves image upsampling and mask adjustment of the nuclear surface. The segmented nuclei can be used for further analysis and quantification of specimens. The individual steps of the process are described in detail in the “Methods” section.

All key parameters used in the segmentation (nuclei sphericity, solidity, volume ) and cell nuclei splitting (nuclei size after distance transform) were established empirically based on the training set (10 images with 520 nuclei). The segmentation accuracy in the test set (JI and F1-Score), related to changes of parameters were shown in the Tables 1, 2, 3 and 4.

**Table 1:** Estimation final value of $NC_{SpherThr}$

$NC_{SpherThr}$	0.65	0.75	**0.85**	0.95
JI	0.826	0.826	0.826	0.826
F1-score	0.914	0.926	0.929	0.929

The best result in bold

**Table 2:** Estimation final value of $NC_{SolidThr}$

$NC_{SolidThr}$	0.65	0.75	0.85	**0.95**
JI	0.829	0.828	0.829	0.827
F1-score	0.800	0.844	0.869	0.888

The best result in bold

**Table 3:** Estimation final value of $NC_{VolMin}$

$NC_{VolMin}$	5e−4	5e−5	**5e−6**	5e-7	5e-8
JI	0.819	0.825	0.826	0.826	0.826
F1-score	0.866	0.910	0.929	0.928	0.928

The best result in bold

**Table 4:** Estimation final factor value of $dist_{MinTh}$

$dist_{MinTh}$	0.3 $dist_{MaxTh}$	0.4 $dist_{MaxTh}$	**0.5**$dist_{MaxTh}$	0.6 $dist_{MaxTh}$	0.7 $dist_{MaxTh}$
JI	0.826	0.826	0.826	0.826	0.828
F1-score	0.920	0.925	0.929	0.923	0.904

The best result in bold

### Examples of cell segmentation results

**Fig. 2:**
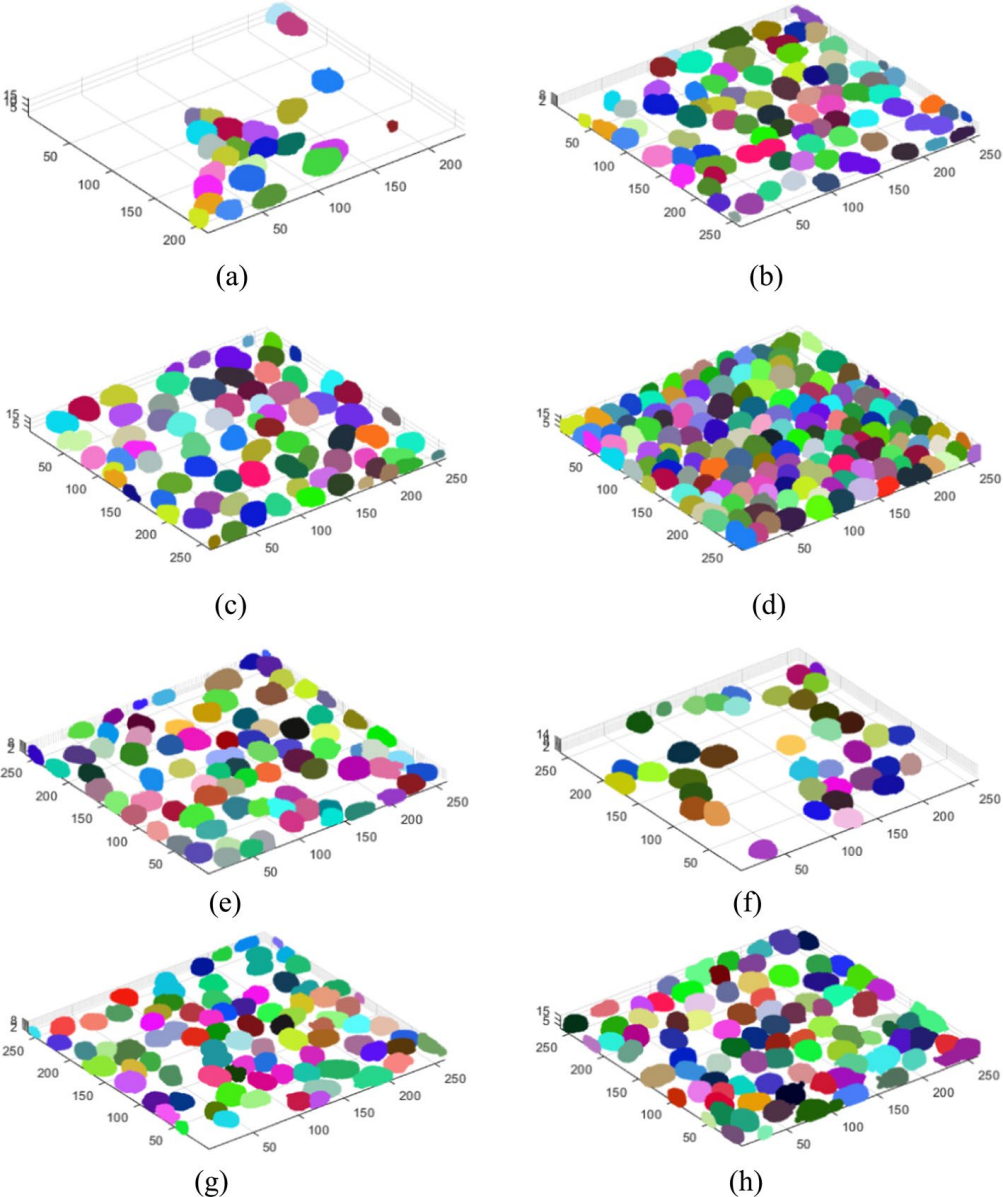
Segmentation process, comprehensive sample results. The cases in** a**–**d** are the specimens from Fig. 6 a–d, respectively.

Figure 2 below visualizes the results of segmentation of selected specimens using the above-described method. The cases in Fig. 2a–d are the specimens from Fig. 6a–d, respectively. The other cases are the most interesting images from the examined set.

When analysing the images, it can be observed that despite the different visibility of cells in the individual layers of the 3D image (cases from Fig. 6), the different confluency of specimens, and the different sizes of single nuclei, the algorithm eliminates the background completely and correctly segments individual cells.

### Segmentation of 3D stacks

**Fig. 3:**
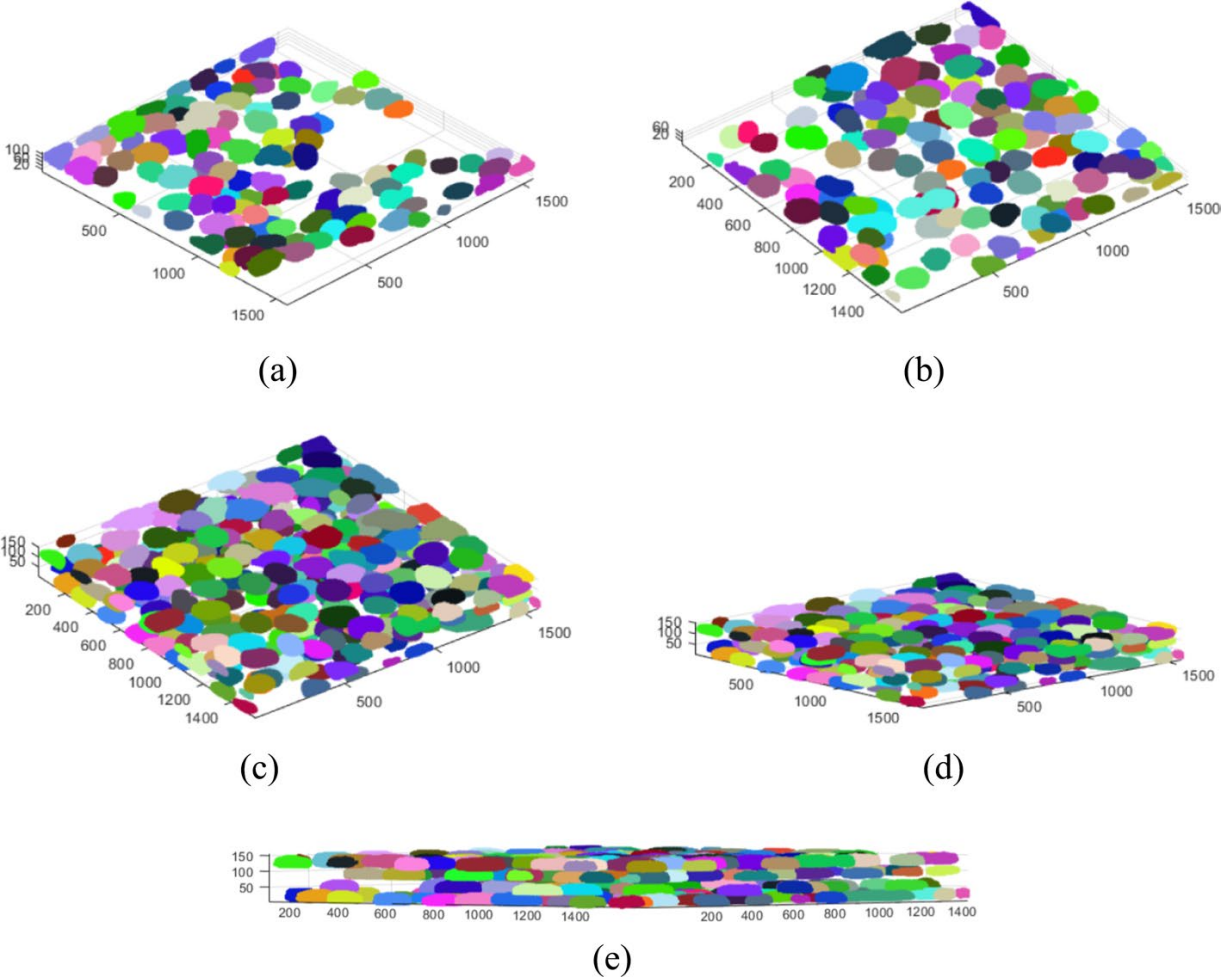
Segmentation results for stacks S1 -** a**, S2 - **b** and S3 -** c**–**e**. Each stack is a result of concatenation of selected stacks from the test set

**Fig. 4:**
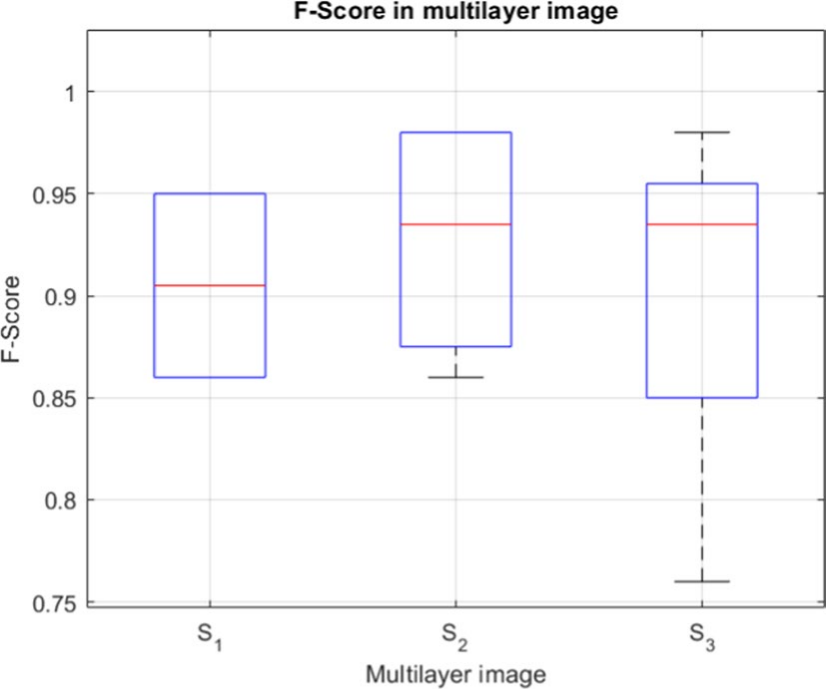
Accuracy in 3D stacks

As the tested 3D images represented monolayer specimens, additional tests were carried out to check the effectiveness of the proposed method in specimens with a greater number of cells and optical layers. For this purpose, we montaged three 3D stacks: a) $S_1$, comprising two 3D images (114 layers—#1, #2); $S_2$, comprising two other 3D images (73 layers—#11,#12); and $S_3$, comprising the $S_2$ stack and two additional 3D images (153 layers in total—#10–#13) ($S_3$). The stacks were merged along the *Z*-axis. Figure 3 shows the results of 3D segmentation of such combined 3D stacks. It can be seen that despite the high cell confluency, the differentiated arrangement of cells in the layers, and the different parameters of the 3D images, the segmentation proceeds in a similar way to single monolayer images. Background elimination is as effective as in the case of monolayer specimens, and the clusters of nuclei of specimens with different parameters do not interfere with the segmentation of the entire 3D stack (Figs. 3, 4).

Table 5 below compares the results of analysis of individual (3D image) specimens with the results obtained after combining them in the *Z*-axis (3D stack). The accuracy metrics for the analogous layers in the case of a 3D image and a 3D stack are similar. The slight differences in these metrics that we observed may be due to differences in the global image characteristics in the 3D images vs. the 3D stacks comprised of these 3D images. These differences result from the different capture conditions of individual images, the different cell types in the comprising 3D images, or the confocal microscope settings during image acquisition. When combining different 3D images in the examined artificial 3D stack, very bright (from one component image) and darker (from the other component image) cells appeared; this is especially noticeable in the case of the greater decrease in effectiveness for image id = 13, which was combined into a 3D stack from 4 real 3D images (153 layers—$S_3$) (Table 5, example 3). As the algorithm tries to automatically determine the operating parameters based on the statistical data of the entire 3D image, in the case of artificial “mismatched” images, the obtained results may be slightly worse, although the values in the real images are better than for the reference methods.

**Table 5:** Results of segmentation of the artificial 3D stack specimens from Fig. 3

Image id/type		Confluency	TP	FP	FN	Prec	Sens	GT	Alg Cells	F1-score	$JI_{Avg}$
								Cells	Count		
								Count			
#1	3D image	Low	23	2	0	0.92	1	23	25	0.95	0.79
#2	3D image	Low	86	2	26	0.97	0.76	112	88	0.86	0.79
$S_1$	3D stack	Low	22	1	1	0.95	0.95	23	23	0.95	0.79
			86	2	26	0.97	0.76	112	88	0.86	0.79
#11	3D image	Low	39	0	1	1	0.97	40	39	0.98	0.85
#12	3D image	Low	78	2	16	0.97	0.83	94	80	0.89	0.84
$S_2$	3D stack	Low	39	0	1	1	0.97	40	39	0.98	0.85
			76	5	18	0.94	0.80	94	81	0.86	0.83
#10	3D image	Low	77	1	8	0.98	0.90	85	78	0.94	0.85
#11	3D image	Low	39	0	1	1	0.97	40	39	0.98	0.85
#12	3D image	low	78	2	16	0.97	0.83	94	80	0.89	0.84
#13	3D image	Mod	88	3	6	0.96	0.93	94	91	0.93	0.84
$S_3$	3D stack	Low/	79	0	6	1	0.92	85	79	0.96	0.84
		Mod	37	1	3	0.97	0.92	40	38	0.95	0.86
			70	7	24	0.90	0.74	94	77	0.81	0.80
			54	3	40	0.94	0.63	94	57	0.76	0.69

### Comparison with other methods

Table 6 summarises the results for the all compared methods. These are the average results obtained from the entire set of 27 3D images. It is worth noting that the proposed method achieves better results for almost all of the measured parameters. The LSetCelTrk method [16] achieves a lower JI, but it works many times faster. Farsight [25] is slightly slower but the results are much better compared to method [11], which yielded the best results among the reference methods.

To perform nuclei segmentation with QCANet, voxel dimensions in our images were adjusted to those in images the QCANet was trained with. Since voxels in the QCANet training images measured either 0.8 $\upmu$m $\times$ 0.8 $\upmu$m $\times$ 1.75 $\upmu$m or 0.8 $\upmu$m $\times$0.8 $\mu$m $\times$ 2.00 $\upmu$m, a arbitrary downsizing factor of 0.15x was applied to X-Y planes. After voxel adjustment, nuclei in our images had approximately the same X–Y–Z sizes as those in the QCANet training images. The downsized stacks were segmented by the QCANet and the returned 3D nuclear masks subsequently upscaled to match the original voxel dimensions and to compare with ground truth. To evaluate 3DCellTracker, X–Y planes in our original stacks were downscaled to 315 $\times$ 315 pixels in order to match the pixel size of images used to train the 3DCellTracker. The specification of 3DCellTracker requires also that the input stack comprises 21 layers. Hence,we further interpolated the input stack along Z axis to generate the 21 layers for sequential processing by the 3DCellTracker. The 3D nuclear mask reconstructed from the outputted 2D masks was then upscaled back to the original resolution to measure nuclei segmentation accuracy against the ground truth.

When tested on our data, the QCANet DL model [27] separated properly all nuclei. The DL model from the 3DCellTracker [28] performed similarly to QCANet. Its average accuracy was lower in high confluency preparations when compared to preparations with low or moderate cell confluency. To segment nuclei, QCANet required GPU, whereas the model from the 3DCellTracker utilized CPU.

The proposed algorithm obtains better results and is also faster than most of the referenced methods. The discussed algorithm does not require any user intervention in order to select or tune the operating parameters as they are determined automatically on the basis of statistical information obtained from the tested 3D images. Table 7 additionally presents the comparison of segmentation results, taking into account the specimen confluency. Segmentation results of tested DL methods are presented in Fig. 15. The results of other methods are shown in the article [11].

**Table 6:** Comparison with other methods (mean values in the test set)

Method	Precision	Recall	F1-score	JI	Time [min]
3D-RSD [11]	0.952	0.847	0.894	0.833	3.5
LSetCelTrk [16]	0.922	0.812	0.858	0.934	152.2
Farsight [25]	0.697	0.798	0.732	0.860	0.8
H-minima shape marking [26]	0.914	0.815	0.858	0.817	2.0
QCANet [36]	0.889	0.740	0.801	0.804	1.9
3DCellTracker [37]	0.971	0.507	0.639	0.631	0.9
**Proposed method**	**0.978**	**0.887**	**0.929**	**0.826**	**1.5**

The best result in bold

**Table 7:** Comparison with other methods (with respect to specimen confluency)

Method	Precision		Recall		F1-score	
	Low–moderate	High	Low–moderate	High	Low–moderate	High
3D-RSD [11]	0.942	0.963	0.830	0.864	0.880	0.909
LSetCelTrk [16]	0.936	0.909	0.862	0.763	0.893	0.824
Farsight [25]	0.751	0.643	0.825	0.771	0.778	0.686
H-minima shape Marking [26]	0.898	0.931	0.795	0.836	0.838	0.878
QCANet [36]	0.903	0.856	0.748	0.722	0.809	0.782
3DCellTracker [37]	0.965	0.983	0.554	0.394	0.692	0.512
**Proposed method**	**0.974**	**0.989**	**0.896**	**0.864**	**0.932**	**0.921**

The best result in bold

## Discussion

In recent years, the number of 3D cell image segmentation methods has been growing rapidly. However, the efficiency and precision of delineating cells and their shapes continues to be a big challenge, especially in direct 3D analysis. High segmentation speed with sufficiently high efficiency is difficult to achieve and requires further development of new methods. Another obstacle is the large diversity of tested specimens. The proposed method, owing to the initial image scaling and the final reconstruction of the segmented cells to the real resolution, makes it possible to maintain high efficiency whilst also reducing the analysis time. Compared to other methods, it combines the global approach with the local one. At the initial stage of the analysis, it relies on the global characteristics of the image to eliminate the background; then, after obtaining the approximate shapes of the cells, it uses local parameters for the separation of cell groups and final shape adjustment. The most important element in the first stage is the method of background elimination and the initial segmentation of cell nuclei that was proposed by the authors on the basis of the combination of variable global and local thresholds (for the determination of various cluster of nuclei) and the shape parameters of nuclei in 3D cluster of nuclei. The algorithm’s operating parameters are determined automatically on the basis of statistical information obtained from the examined 3D image. In the second stage, the most important element among the proposed solutions is the method of generating seeds of single cell nuclei and splitting groups of cells into single cells. Combining the results obtained from the modified 3D distance transform and the 3D seed watershed algorithm allows additional verification of the number of cells that result from splitting. The size range of normal nuclei is determined automatically based on the size of the examined 3D image and the size of the analysed clusters of nuclei subjected to segmentation. For faster operation, background elimination and cell group splitting are performed on images with reduced sizes in the *X*-axis and the *Y*-axis. Finally, the cell nuclei are reconstructed after segmentation. The final shape of each reconstructed cell is corrected at 1:1 scale, taking into account the information about the intensity level inside each detected cell. As a result, surface inaccuracies resulting from image scaling are significantly reduced. The developed method works very well for specimens of low, medium, and high confluency (Table 7) and is able to divide large clusters of nuclei consisting of several dozen adjacent cells (examples in Figs. 6d and 12b, c). However, the average F1-score and JI achieved by the 3DCellTracker were much lower than those achieved by the QCANet because the 3DCellTracker performed poorly on in specimens with high confluency. Both DL models had overall worse performance than the proposed method (Tables 6 and 7). We reason that the proposed method worked better than DL models because the DL models are sensitive to spherical object shapes that they were trained with. Our data however, includes more arbitrary nuclear shapes (spherical, ellipsoid and half-moon) arising from experimental conditions.

The further improvement in performance may be related to the acceleration of the final shape adjustment algorithm, which, with a large number of cells, has a visible impact on the total analysis time. At the present stage, this algorithm does not use parallel computations, which has a large impact on its duration. For better verification of its effectiveness, it seems reasonable to examine the segmentation effectiveness using ground-truth data prepared in the form of 3D masks.

## Conclusions

The proposed method allows fully automatic segmentation of 3D cell specimens obtained from a confocal microscope. All processing steps are carried out in 3D, which makes it possible to eliminate the typical problems that occur during 2D segmentation (e.g. not taking into account information from adjacent layers). The obtained results, compared to other methods, demonstrate higher precision, specificity, F1-Score and a comparable JI (Table 6). In comparison with DL methods, the proposed method works faster (images were downsized to achieve similar voxels sizes), achieves better results in JI, F1-Score, precision and other parameters. The duration of the proposed method is comparable or shorter than that of the reference methods (and when the final shape adjustment block is excluded, it is additionally shortened at the expense of a slight reduction of the JI). The advantage of the method is greater speed and efficiency compared to other methods and the use of global and local features at various stages of the algorithm’s operation. The proposed algorithm can reliably segment nuclei in real and simulated large stacks of 3D images of cells with various degrees of cell confluency. Owing to the local capture of image characteristics and the multiscale nature of 3D image processing, our algorithm can satisfactorily cope with a variety of cell types, changes in nuclear morphology induced by cytotoxic drugs, and decreased fluorescence along the 3D stack.

## Methods

### Dataset

For this project, we reused a set of previously collected high-resolution image data. The method was developed using an independent set of 10 3D images (containing 521 nuclei). For testing we used a separate set of 27 (from #1 to #27) 3D images (with 2,367 nuclei). These images were acquired to investigate the expression of the 5-methylcytosine marker in cancer cell lines treated with chemotherapeutics that inhibit DNA methylation. The set includes image z-stacks of treated and untreated cells of DU145 human prostate carcinoma, and treated and untreated HuH-7 carcinoma of the liver fixed on glass slides. Besides the 5-methylcytosine marker, the cells were counterstained with 4’,6-diamidino-2-phenylindole (DAPI)—a common blue-fluorescent dye that binds to DNA. Staining was followed by imaging with a confocal laser-scanning microscope (TCS SP5 X Supercontinuum, Leica Microsystems Inc.). The imaging yielded z-stacks with 35–50 high-resolution 1576 $\times$ 1576px large serial optical sections with a voxel size of 120 nm $\times$ 120 nm $\times$ 250 nm (*X*-, *Y*-, and *Z*-axis) and 12 bit/px fluorescence intensity for each stain. DAPI and 5-methylcytosine signals were recorded in separate channels. For the numerical experiments described in this paper, we repurposed a set of 27 DAPI *Z*-stacks that represented DU145 cells and HuH-7 cells of low (up to 40 nuclei), moderate (41–65 nuclei) and high confluency (73–190 nuclei), and with a high variability of nuclear staining intensity and texture [11]. The mid-optical section in each stack is supplemented with ground-truth delineation to assess the segmentation performance. All 2367 nuclei in the whole set of 3D images were outlined manually by an expert.

**Fig. 5:**
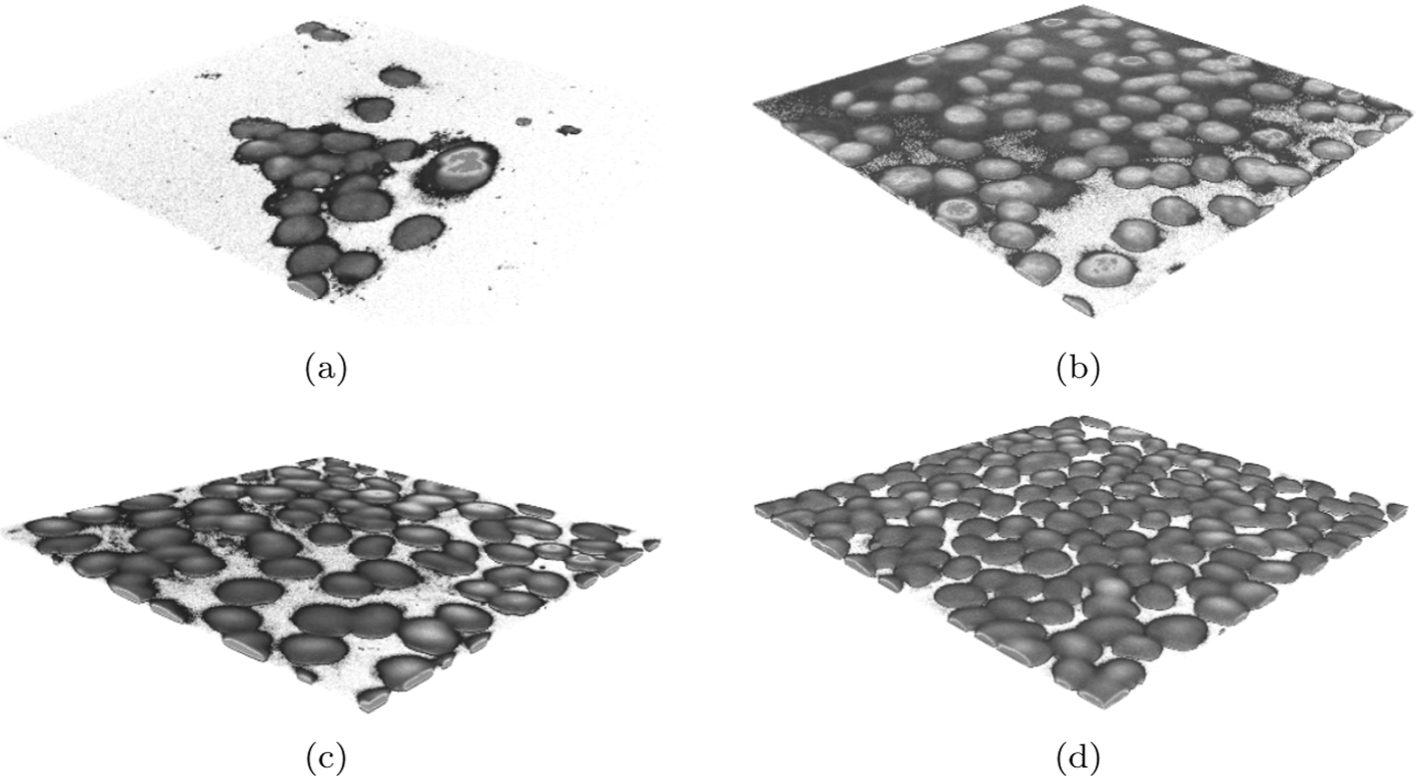
Examples of images with a manually selected brightness threshold, and the common problems that occur in the specimens. The cell confluency varies from low, through medium, to high (a–d)

The specimens were very diverse: some of them showed an inhomogeneous background and disturbances in the outermost layers, thus making it difficult to separate cells from the background (Fig. 5b–d). Figure 5a additionally shows the obscuration that appears in some specimens, which makes it difficult to delineate the precise shape of cells. The direct application of a local or global threshold value for the whole specimen and all cells nuclei does not make effective separation of cluster of nuclei possible in all tested specimens. Two types of artifacts can be distinguished in the tested specimens: local noise in individual image layers as a result of the imaging method and imaged cell types, Fig. 5a; directional noise with a specific trend in the samples along the *Z*-axis, Fig. 5b, and in layers that are not near the microscope objective, Fig. 5c. In such cases, even manual setting of the background intensity threshold to simultaneously separate all cells from the background without affecting the nuclear shape to a great extent is difficult or even impossible. The proposed method allows these problems to be eliminated, as shown in Fig. 5. This is possible because the threshold values are initially selected in two stages: globally for the entire specimen and then locally for the groups of cells, analysed separately. As the noise level in different areas of the specimen varies, the visibility of the cells is different. The second stage consists in performing proper 3D segmentation of the groups of cell nuclei that are formed after the background elimination. Since not every segmented cluster of nuclei is correct (i.e. it is not a single cell nucleus) and groups of adjacent cells nuclei appear in the image, it is necessary to divide such nuclei groups. The 3D watershed method, the analysis of the results from the modified 3D distance transform, as well as the 3D morphometric parameters of the cluster of nuclei were all used in the cell splitting procedure.

**Fig. 6:**
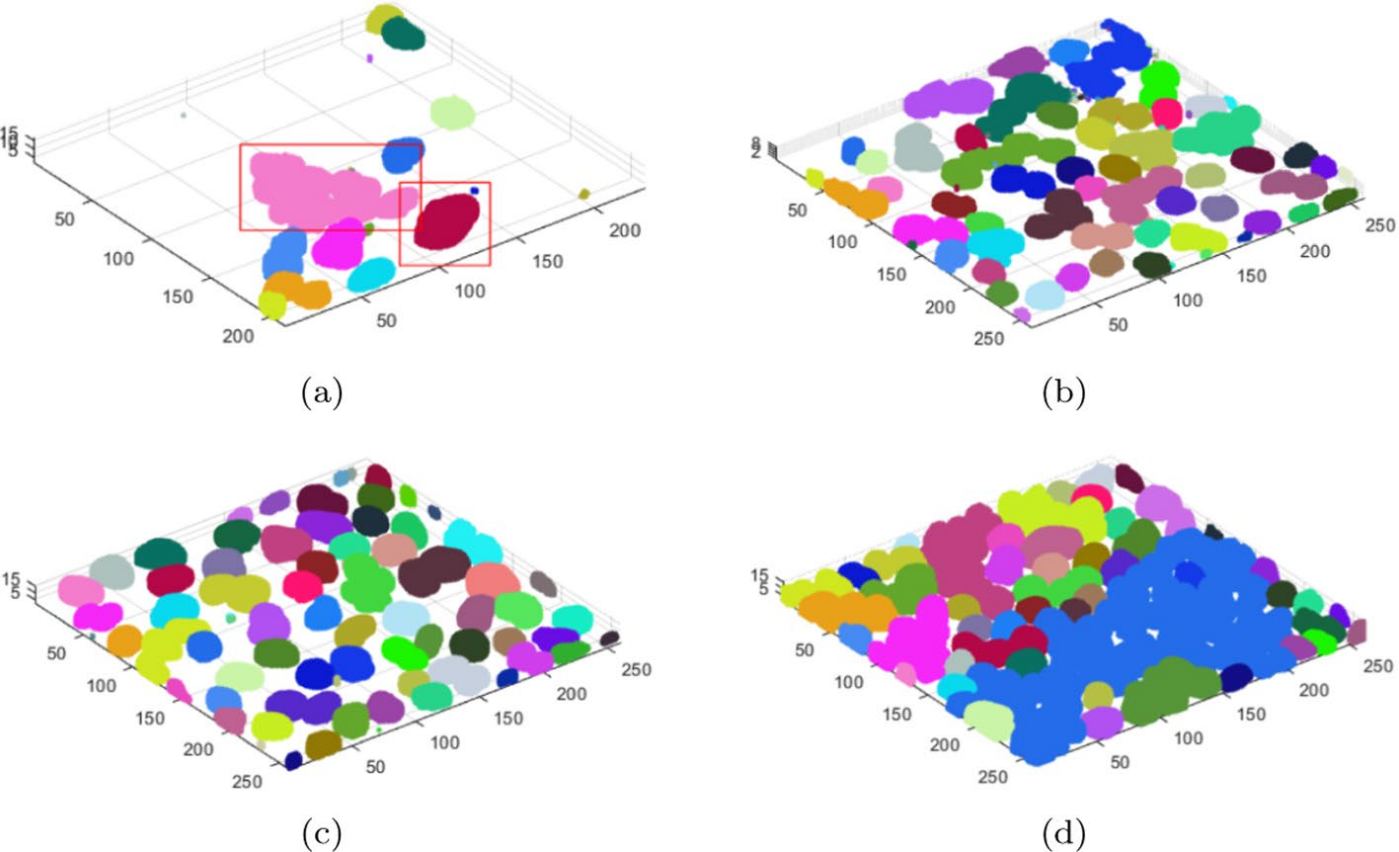
Examples of background elimination and cluster of nuclei separation for specimens with different confluency (the cases from Fig. 5a–d, respectively)

The results of background elimination and cluster of nuclei segmentation for the examples from Fig. 5 are presented in Fig. 6. The selected examples show that cell confluency varies from low, through medium, to high (Fig. 5a–d). It can be seen that the background was eliminated correctly in each case. In some cases, after the background elimination, the actual single nuclei of cells as well as groups of adjacent cells are immediately separated. In Fig. 6d, after removing the background, a very large cluster of nuclei containing several dozen cells is formed and requires separation. Small areas of residual background (e.g. Fig. 6a) will be removed at a later stage. Voxel clouds surrounding some cells (Fig. 5a) are also removed in the background elimination step. Figure 6a shows some selected groups of adjacent cells, the segmentation of which is discussed below and presented in Fig. 11 in the Segmentation and separation of cell groups section.

**Fig. 7:**
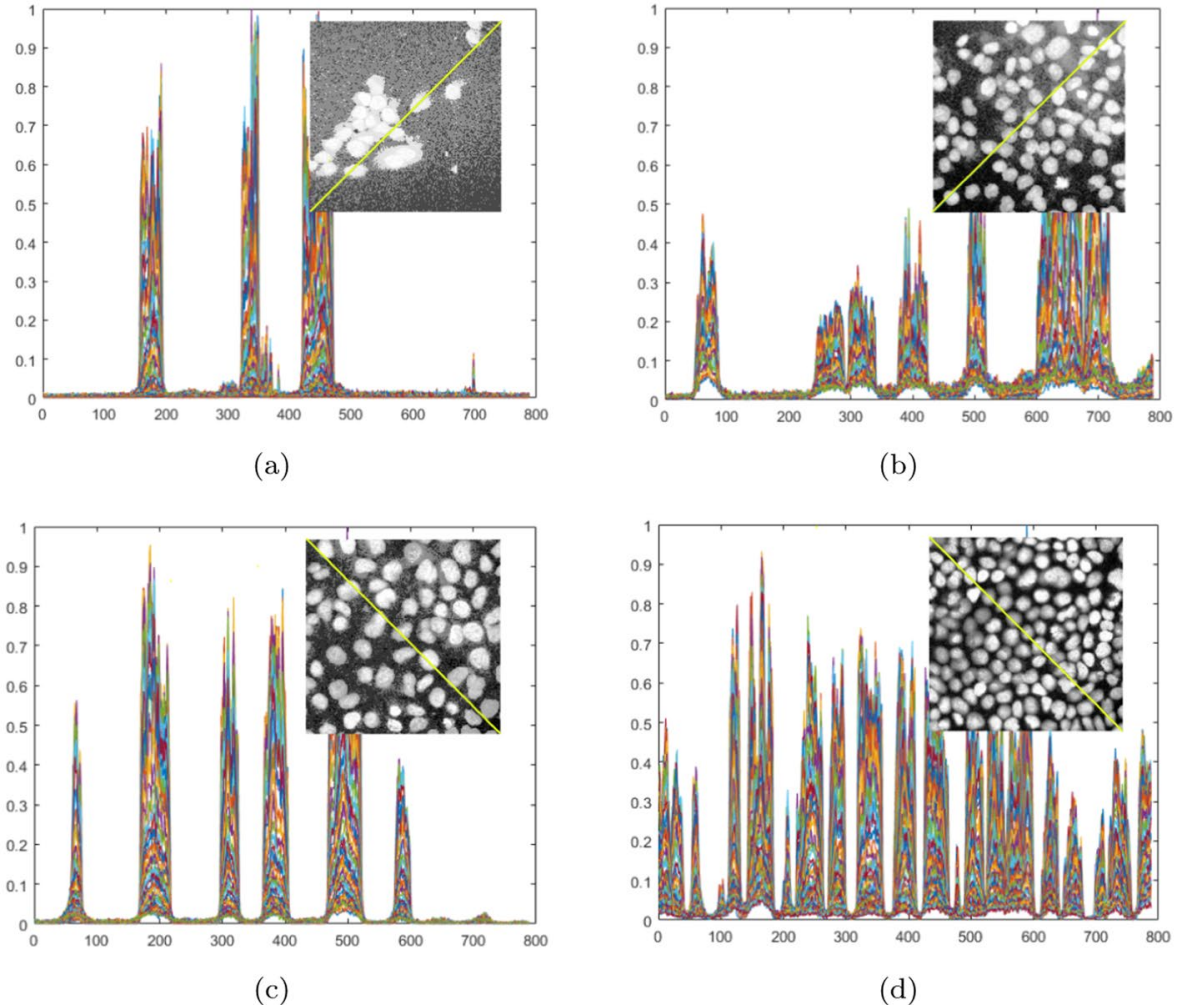
Examples of layers with noise and oblique profiles for all layers in the specimens. The noise level for specimens from Fig. 5a–d), respectively

Figure 7 presents the above-discussed problems (related to several types of image disruption) using the profiles of the examined images. For the examples in Fig. 5, oblique profiles were made, which show how the cells in successive layers stand out against the noise. In order to better present the level and type of noise in the images, the histogram was aligned. Figure 7a shows the noise over the entire image area. In Fig. 7b, noise appears in the vicinity of cells and is characterized by a trend similar to the profile direction. In the case of Fig. 7c, the noise level increases again in the upper right part of the image. In Fig. 7d), the noise is relatively low, and the cells are clearly visible. At the same time, it can be observed that the noise level in most of the presented images is not equal across the entire profile (Fig. 7b–d).

### Background elimination and nucleus segmentation

**Fig. 8:**
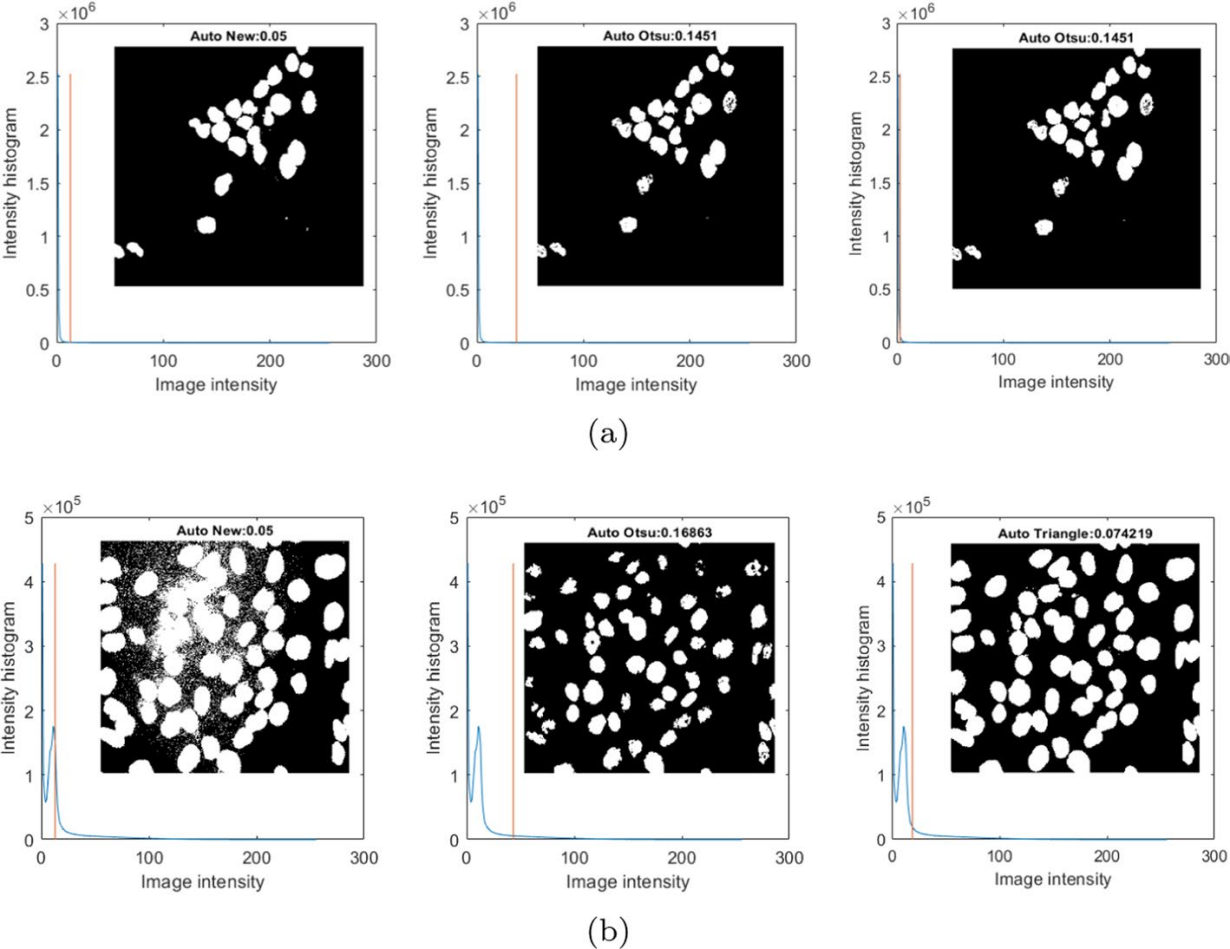
Method for determining the background threshold based on the image intensity histogram, and comparison of the results of different methods for the selected layer. The results for two selected specimens (**a**,** b**)

The first step is to prepare the images for segmentation. Due to the large size (1576 $\times$ 1576 px) and the several dozen layers in the *Z*-axis, compared to the originals the size of the examined images was reduced by 50% (in the X and Y axes to 788 $\times$ 788 px) using the nearest neighbour method. The image size in the *Z*-axis remained unchanged. Initial scaling can reduce the analysis time and concentrate the nucleus voxels in the outermost layers, where their cluster of nuclei is significantly rarefied and may be poorly visible. Due to the large variation in the brightness and dynamics of images, scaling makes it possible to improve the visibility of cells by averaging the intensity values of voxels inside the cells. The thus-prepared images are then converted into a $M_{3\times {3}\times {3}}$ sub-matrix sized 3$\times$3$\times$3 voxels. This procedure enables analysis of the voxel and its surroundings in the examined image. Subsequently, each $M_{3\times {3}\times {3}}$ is classified as background or cellular structure (cell nucleus or a cluster of nuclei). This classification is based on a quick thresholding operation with an automatically determined global threshold. However, it proved to be impossible to establish a universal global threshold value that would effectively separate all cells from the background in all images. During the trials, it was observed that an experimentally selected threshold value of $Th_{Int1}$ = 5% of the maximum intensity $I_{Max}$ (from the 3D matrix of the $Im_{3D}$ image) provided good background elimination and cell separation results in the examined images (in the case of 6 images, the nuclei of cells were correctly segmented in the first stage). For the other 21 images, this value was not optimal (too many groups of adjacent cells were formed) and further splitting was necessary. As a result of comparing the effectiveness of various threshold values ($Th_{Int}$ from 5%, 10%, 15% to 20%), it was also established that a value of $Th_{Int2}$ = 10% $I_{Max}$ is the maximum value that allows effective separation of nuclei without destroying their structure, which would prevent further segmentation. Therefore, it was assumed that the threshold value used for background elimination should not exceed 10% of the maximum intensity in the image. In order to determine the initial value of the global threshold, $Th_{Int1}$, for the classification of cell and background voxels (represented by $M_{3\times {3}\times {3}}$ sub-matrices), several selected methods were tested, including our own, for which the algorithm yielded the best results. Figure 8 shows the results of background elimination and binary mask delineation for a number of selected automatic threshold determination methods based on the image histogram. Complex methods that combine several basic methods were also tested in the noise elimination process, including anisotropic diffusion and background subtract rolling ball algorithms [33]. However, it turned out that their computational effort was significantly greater, and they had no influence on the obtained results. Therefore, we proposed our own method of determining the initial value of the global threshold, $Th_{Int1}$, to eliminate noise and background. This method is based on the assumption that the noise in the examined images is within a certain range of values (Eq. 1). On this basis, the image brightness range is determined in which the number of noise voxels is the highest. This method yielded very good results in most images; it effectively responds to the noise trends in the image by increasing the value of the threshold, $Th_{IntM1}$. In the case of some images, it was impossible to further increase the threshold without damaging the nucleus structure. As a result of further tests, it was confirmed that the use of the triangle thresholding method [34, 35] made it possible in these cases to determine a greater threshold value without destroying the nucleus structure, $Th_{IntM2}$, as shown in Fig. 8.

1 $$\begin{aligned} V_{Th}(x,y,z)_{(i)}&= \left\{ \begin{array}{ll} 1 &{} \quad if\,\,Th_{(i)}-N_{Level}< Im_{3D}(x,y,z) < Th_{(i)}\\ 0 &{}\quad otherwise \end{array} \right. \\ VoxCount_{(i)}&= \sum V_{Th_{(i)}} \\ Th_{Int1M1}&=max (VoxCount_{(i)}) \end{aligned}$$

where:

- $N_{Level}$ (0.05) is the signal-to-noise level in the examined set of images [3, 36]
- *X*, *Y*, *Z*—dimensions of the 3D image along *X*,*Y*,*Z* axes
- *x*, *y*, *z*—voxel coordinates in 3D in the ranges $x= 1,\ldots ,X$ , $y= 1,\ldots ,Y$, $z= 1,\ldots ,Z$
- $Im_{3D}$—is the examined 3D image
- $Th_{(i)}$ $\in$ {0.05 ...0.2} are the successive values of the threshold for which background voxels are counted

As a result, both methods were combined and a solution was adopted in which $Th_{Int1}$ is determined as the maximization of the results of two automatic threshold determination methods (Eq. 2), i.e. the method proposed by the authors (Eq. 1) and triangle thresholding [34, 35].

2 $$\begin{aligned} Th_{Int1} = max([Th_{IntM1} , Th_{IntM2}]) \end{aligned}$$

where:

- $Th_{IntM1}$—threshold determined by the method proposed by the authors (Equation 1)
- $Th_{IntM2}$—the threshold value equals 10% $I_{Max}$

Automatically computed $Th_{Int1}$ values (i.e.about 5%, 6%, 8%, 10%) depend on the image content. In case of some images, the computed $Th_{Int1}$ allows to fully segment the image and the algorithm does not reach the second stage which requires computing $Th_{Int2}$ . After determining the initial $Th_{Int1}$ value, the algorithm starts image segmentation with the $Th_{Int1}$ * $I_{Max}$ value; it then eliminates the background and detects cluster of nuclei (Eq. 3). At the same time, the resulting matrices $M_{InitResult}(x,y,z)$ that represent the intensity values are created for each $M_{Result}(x_R,y_R,z_R)$ element which is a cell nucleus or cluster of nuclei.

3 $$\begin{aligned} M_{3x3x3} (x_R,y_R,z_R )&= mean(Im_{3DSc} (x,y,z)) \\ M_{Result} (x_R,y_R,z_R )&= \left\{ \begin{array}{ccc} 1 &{} if &{} M_{3x3x3} (x_R,y_R,z_R ) > I_{Max} * Th_{Init}\\ 0 &{} &{} otherwise \end{array} \right. \end{aligned}$$

where:

- $Th_{Init}$ can take values in the range 0-1
- $I_{Max}$—maximum brightness in the 3D image

Next, each segmented cluster of nuclei is checked for size and shape as well as the possibility of its further division; it is then classified as $NC_S$ or $NC_G$. The assessment criteria for these cluster of nuclei are described in Eq. (4):

4 $$\begin{aligned} NC_{Solid}&= \frac{NC_{Vol}}{NC_{VolConv}} \ge NC_{SolidThr} \\ NC_{Spher}&= \frac{ \root 3 \of { 36 * \pi * {NC_{Vol}}^2}}{NC_{Surf}} \ge NC_{SpherThr} \\ NC_{VolMin}&\le NC_{Vol} < NC_{VolMax} \end{aligned}$$

where:

- $$NC_{Vol}$$–3D nuclear volume
- $NC_{VolConv}$—convex hull volume of the 3D nucleus
- $$NC_{Surf}$$—3D nucleus surface
- $$NC_{Spher}$$—3D nucleus sphericity
- $NC_{SolidThr}$—threshold value of 3D solidity = 0.95
- $NC_{SpherThr}$—threshold value of 3D sphericity = 0.85
- $NC_{VolMin}$ and $NC_{VolMax}$—min and max values of the 3D nuclear volumes in the training set

We characterized the nuclear size and shape by sphericity, solidity and volume which are properties of ellipsoid [37, 38]. To adjust $NC_{SolidThr}$, $NC_{SpherThr}$, $NC_{VolMin}$ values , we measured JI and F1-score after substituting values from a wide range. The final values for $NC_{SolidThr}$, $NC_{SpherThr}$ and $NC_{VolMin}$ were picked when JI and F1-score were the highest (Tables 1, 2, and 3).

Any $NC_G$ segmented structure that does not meet the criteria for a single cell nucleus is reanalysed. This time, the algorithm uses a larger threshold: $Th_{Int2}$ * $I_{Max}$. As a result of increasing the local threshold, groups of cells (undivided in the first stage) are slightly reduced, which improves their separation from one another. The cells are again subjected to splitting. The proposed method uses the variable local threshold value and distance transform applied to the region in the image which contains clusters of cells in order to split them. It improves the efficiency of cell division and segmentation in specimens where the visibility of cells varied greatly or there was noise in a specific direction.

In the case of specimens in which the visibility of all cells was similar, the cells were easy to separate, there was no interference (e.g. in the form of a directional increase in noise level), and $NC_S$ were efficiently isolated by the algorithm in the first step. When large groups of cells were in contact with each other after thresholding with global $Th_{Int1} * I_{Max}$, only the remaining $NC_G$ cluster of nuclei were reanalysed (with an increased threshold of $Th_{Int2} * I_{Max}$). The use of the variable local threshold made it possible to avoid too much reduction of the area of all cells in the specimen and to reduce only densely clustered cells nuclei.

**Fig. 9:**
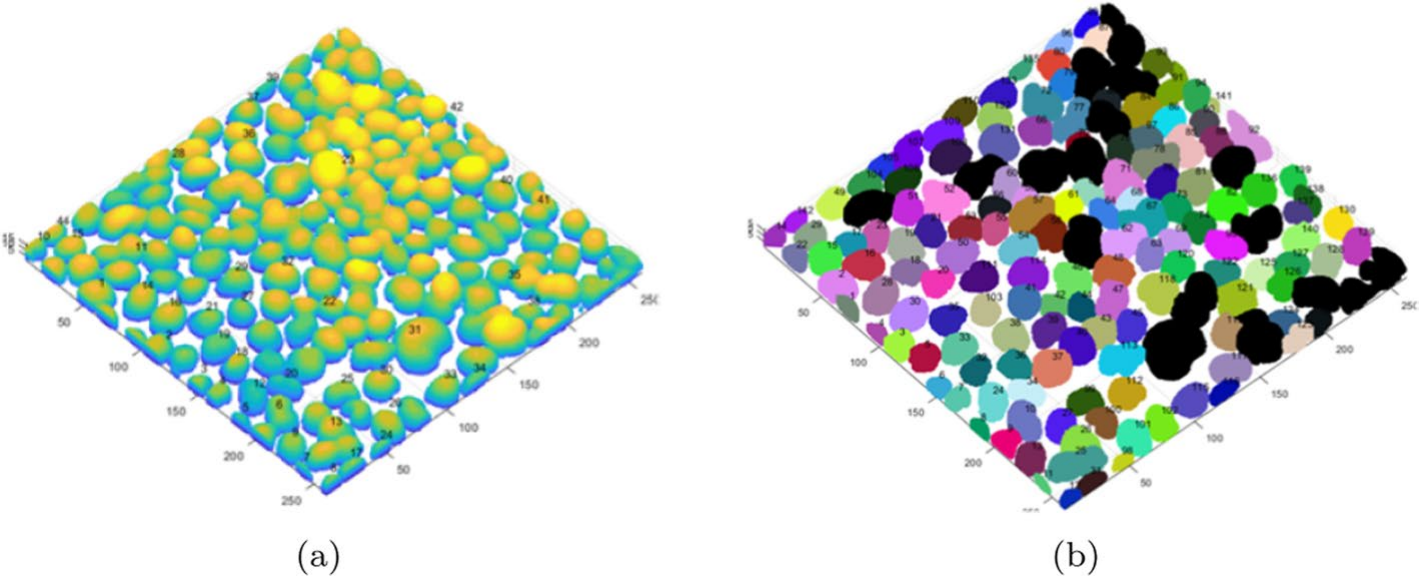
Tested specimen (**a**), single cells nuclei ($NC_S$) correctly separated and groups of nuclei ($NC_G$—in black) transferred for further splitting (**b**)

Figure 9 below shows the case of a high-content specimen for which the algorithm, in the first step ($Th_{Int1} * I_{Max}$ threshold), segments the subset of single cells correctly, and the remaining groups that were not successfully segmented (marked in black) are transferred to the second stage ($Th_{Int2} * I_{Max}$ threshold; Fig. 9b). It can be seen that in the case of high cell confluency, not all cluster of nuclei may be segmented correctly in the first step and may contain adjacent sub-nuclei, as shown in Fig. 9b).

**Fig. 10:**
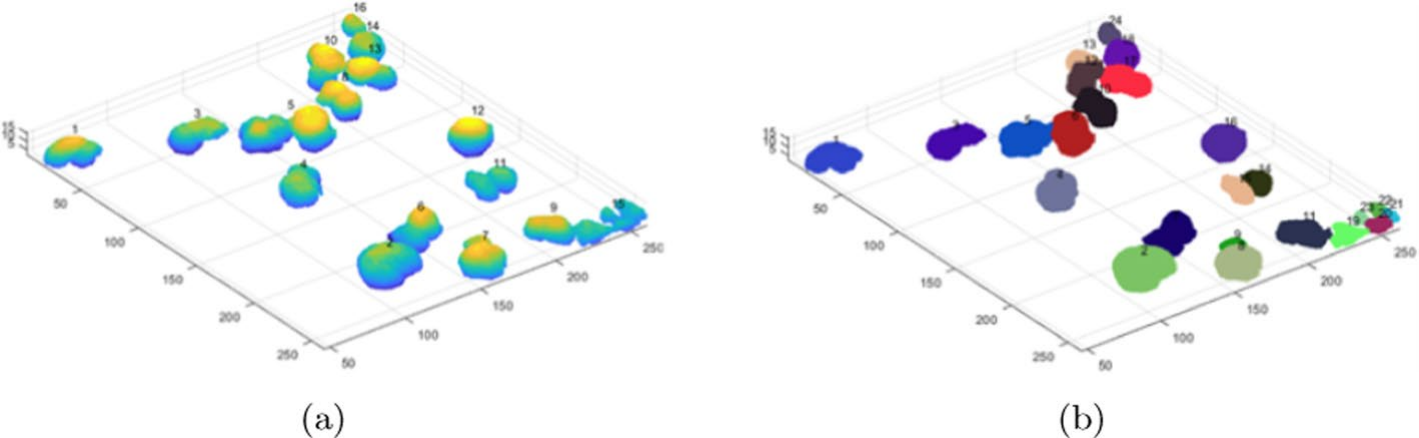
Second step of analysis of the high cell confluency specimen (**a**); division of the remaining $NC_G$ cluster of nuclei from Fig. 9b, marked in black (**b**)

Only the remaining $NC_G$ structures (cluster of nuclei) that are too large and those that could not be separated in the first step (the cases from Fig. 9b, shown in Fig. 10a) are re-segmented (with a higher threshold).

Figure 10 shows the second step of division of the remaining specimen cluster of nuclei with the threshold $Th_{Int2}$. The application of a higher local threshold value reduced the cell shapes, which enabled their separation. The cluster of nuclei of the cells adjacent in the first step (Fig. 10a) are now segmented correctly (Fig. 10b) (marked with different colours).

### Segmentation and separation of nucleus groups

The segmentation in the $M_{Result}$ matrix is performed locally based on the shape, size criteria, and verification of the possibility of division by combining the results of the 3D distance transform [39, 40] and the 3D watershed, according to the following assumptions regarding the parameters of a single nucleus: $NC_S$ and Equation (4). The following parameters, which determine the correct shapes of nuclei in 3D, were designated experimentally on the basis of an additional training set that comprised 10 images with over 520 nuclei (the final values were estimated based on Tables 1, 2, 3, and 4):

- nucleus solidity parameter $NC_{Solid}$, as in Eq. (4)
- nucleus sphericity parameter $NC_{Spher}$, as in Eq. (4)
- nuclear volume parameter $NC_{Vol}$ ranging from $NC_{VolMin}=5*size(Im_{3DSc})*10^{-6}$ to $NC_{VolMax}=2*size(Im_{3DSc})*10^{-2}$, related to the 3D image resolution, as in Equation (4)
- parameter of the size of the separated structure ($im_{Dist23}$) on the basis of the modified 3D distance map according to the relationship: $im_{Dist23}=(im_{Dist2}+2*im_{Dist3})$

The nucleus group division algorithm is based on a modified 3D distance transform ($im_{Dist23}$ matrix, which includes only currently analysed nucleus group) that takes into account the disproportions in the sizes of the images on the *X*, *Y* and *Z* axes. The values of the standard 3D distance transform are significantly reduced by the values in the *Z* axis, which makes it impossible to use this method directly to assess the size of the examined nuclei in 3D.

After separating the cell nucleus structures from the background, the algorithm classifies each structure as a single nucleus or a group of nuclei. Two types of nuclei structures may appear at this stage. The first one is a nucleus that meets the shape and size criteria; it is classified as a single, properly separated nucleus, $NC_S$. The second type is a structure which does not meet the shape and size criteria, $NC_G$. The algorithm attempts to divide it by analysing the results of the 3D distance transform (based on $im_{Dist23}$) and the 3D seed watershed. When the structure is not divided (the number of generated seeds is equal to 1 but the structure is greater than the threshold value, $NC_{Vol}$—Eq. 4); this means that it is a large structure of adjacent nuclei. Then, the entire $NC_G$ cluster of nuclei is subjected to splitting with a higher local threshold, $Th_{Int2}$. The process of cell nucleus division works the same as for $Th_{Int1}$; only the input data differ. These are the nucleus structures separated from the background that were created after applying the local threshold $Th_{Int2}$.

The analysed structure (cluster of nuclei or single nucleus) can be classified as $NC_S$ in four cases:

- in the first stage, when $NC_{Solid} \ge 0.95$, $NC_{Spher} \ge 0.85$ and $NC_{Vol}$ is in the range from $NC_{VolMin}$ to $NC_{VolMax}$;
- when it does not meet point (a) but is not too large and cannot be divided;
- when it is divided into sub- nuclei, all sub-nuclei are added (as $NC_{Single}$);
- when it was not divided in the first stage (at $Th_{Int1}$), or it was too large and was not divided in the second stage (at $Th_{Int2}$) and there is no further possibility of splitting it or increasing the threshold.

**Fig. 11:**
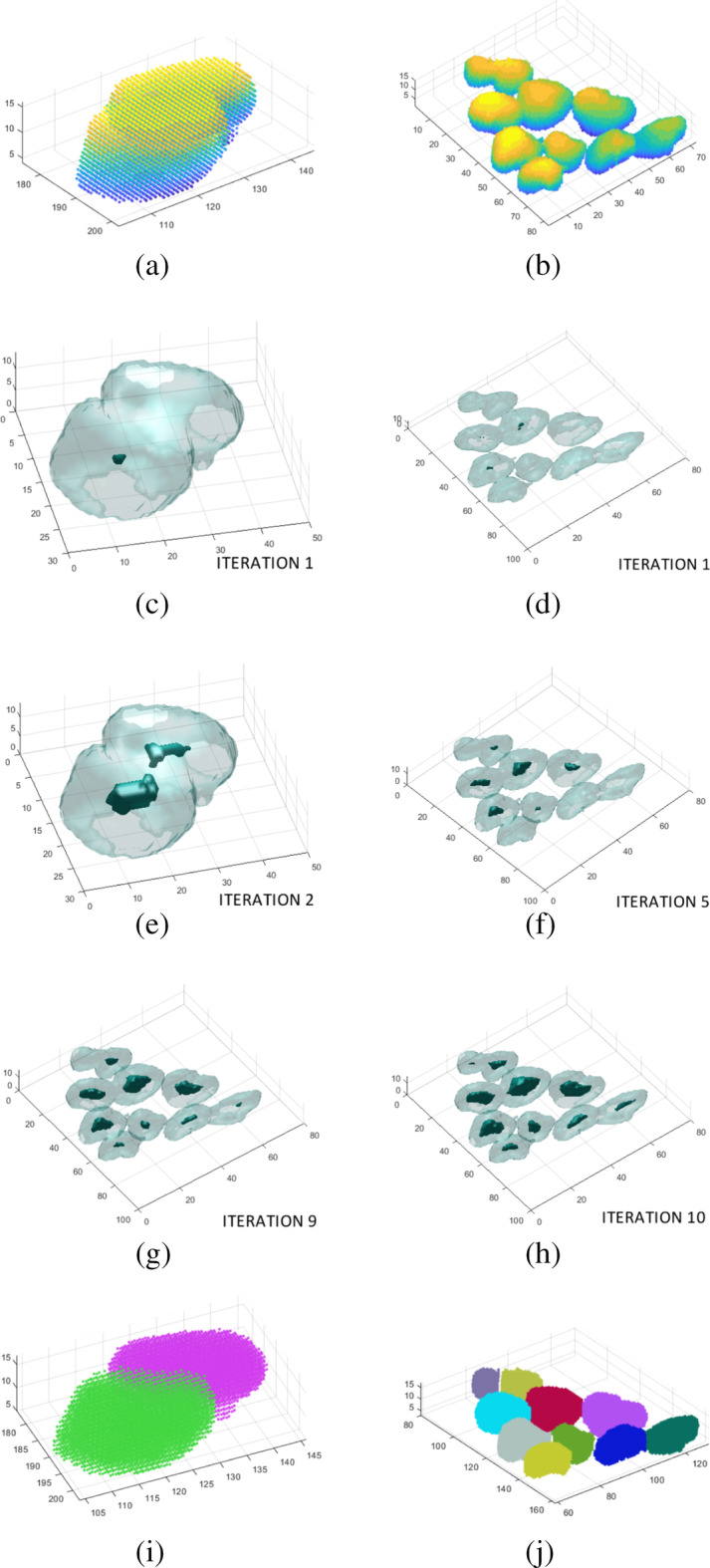
Steps of splitting some selected cell groups from Fig. 6a (**a**,** b**). The selected iterations of the specimen (**a**–**c**,** e**). The selected iterations of the specimen (**b**,** d**–**h**). The results of splitting two selected cell gropus (**i**,** j**)

Figure 11 shows the steps of segmentation of the $NC_G$ to be divided. After determining the value of the modified 3D distance transform ($im_{Dist23}$) in the section of the 3D matrix ($M_{Result}(NC_{GID})$) containing the tested $NC_G$ cluster of nuclei, the acceptable size of nuclei for splitting is calculated with Eq. (6). The performed tests resulted in adopting the limit values that define the minimum and maximum cell sizes (in relation to the value of the 3D distance transform—Eq. 5 for the examined cluster of nuclei—$NC_G$). These values allow the separation of the generated seeds of the created cells that result from the analysis of voxel sets in different cell groups:

5 $$\begin{aligned} dist_{MaxTh}&= max(distanceTransform(M_{Result\_NCG})) \\ dist_{MinTh}&=0.5*dist_{MaxTh} \end{aligned}$$

The analysis of each $NC_G$ cluster of nuclei begins with a threshold value for a modified 3D distance map equal to $dist_{MaxTh}$ and decreasing to $dist_{MinTh}$ (values are determined automatically for each analysed nuclei group—the best factor value is estimated based on Table 4 ). The iteration step was $\Delta _{Dist}=-0.2$ (the fastest analysis rate and nucleus division efficiency).

Figure 11 presents the results of splitting two nucleus groups with different degrees of connection. Figure 11a, b represents the $NC_G$ cluster of nuclei from the example in Fig. 6a (cluster of nuclei marked with a red frame). Both cluster of nuclei are correctly separated from the background, so the algorithm tries to divide them at a later stage. In the next stages (Fig. 11c, d), it can be seen that as the threshold size for $dist_{Th}$ decreases, and new seeds are generated that represent the cell centres within the group. For different cluster of nuclei, the number of iterations necessary to generate seeds is different: Fig. 11c (2 iterations), Fig. 11d (10 iterations).

As the $dist_{Th}$ value decreases in subsequent iterations, the size of the cell seed in the group increases and new ones may appear. Therefore, at this stage it is required to classify each seed as newly created or previously generated (each seed was enlarged in the next iteration). All seeds analysed in each iteration are classified as existing or new ones. The criterion used was the distance between the seed centroids (from the previous and actual iterations) and the sum of the values of the 3D distance map in the centroids of these seeds—Eq. 6. The list of generated seeds is then passed to the 3D seed watershed method, whose task is to separate the voxels of nucleus groups—$NC_G$. Due to such initialization of the 3D seed watershed function, the efficiency of splitting is improved, which is particularly evident in the case of two cells that are very close together (Fig. 11a and the result in Fig. 11e). Figure 11c, d show the seeds generated in subsequent iterations. The results of the last iteration are used to initialize segmentation by the 3D seed watershed. The division of the $NC_G$ cluster of nuclei generates the resulting set of $NC_S$—Fig. 11e–f. The cluster of nuclei from Fig. 11a is split into two nucleus and the cluster of nuclei from Fig. 11b is split into 10 nucleus.

6 $$\begin{aligned} seed_{New} = \left\{ \begin{array}{ll} false &{} \quad if \,\, distCentr_{Act-Prev} < im_{Dist(Act)}+ im_{Dist(Prev)}\\ true &{} \quad if \,\, distCentr_{Act-Prev} \ge im_{Dist(Act)}+ im_{Dist(Prev)}\\ \end{array} \right. \end{aligned}$$

where:

- $distCentr_{Act-Prev}$—Euclidean distance between the seed centroids from the actual and previous iterations
- $im_{Dist(Prev)}$—value of the function of the 3D image distance in the cell centroid in the previous iteration
- $im_{Dist(Act)}$—value of the function of the 3D image distance in the cell centroid in the actual iteration

**Fig. 12:**
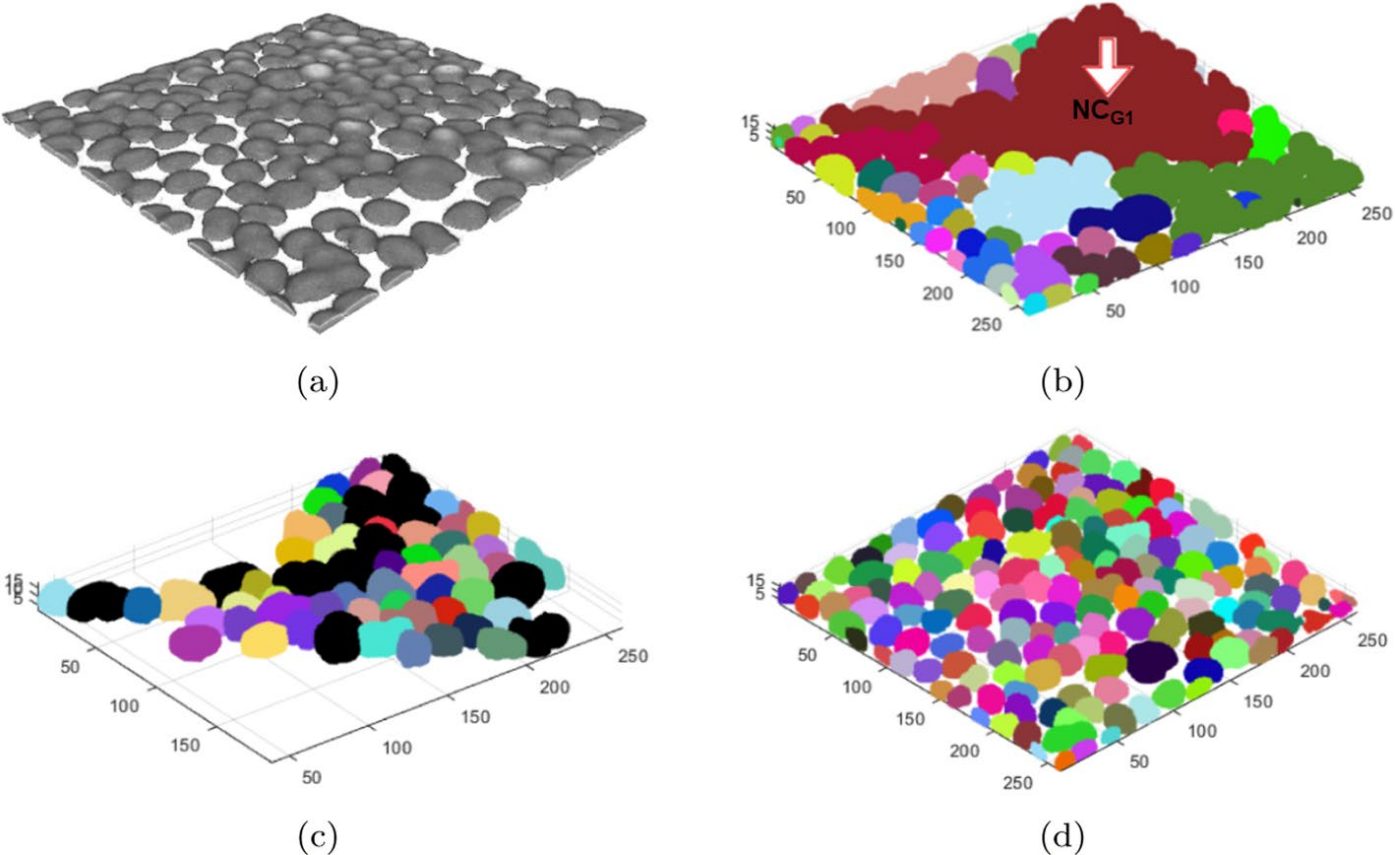
Segmentation of a large high-content complex cluster of nuclei.** a** The selected specimen,** b** first stage of segmentation–with global threshold (**c**) second stage–the segmentation of undivided cluster of nuclei with local threshold (**d**) final result

Figure 12 presents another case of segmentation of a very large $NC_{G1}$ cluster of nuclei (containing several dozen sub-nuclei and several dozen $NC_S$—Fig. 12b). This example shows how effectively the proposed method segments such large cluster of nuclei. Based on the results of the 3D distance transform in the entire $NC_{G1}$, the algorithm automatically determines the size range of the nuclei contained in this cluster of nuclei. It then attempts to divide the high-content $NC_{G1}$ cluster of nuclei. Some nuclei in the specimen are already separated in the first step (for $Th_{Int1}$); the undivided ones (black—Fig. 12c) are again subjected to splitting (after increasing the local threshold to $Th_{Int2}$). Dividing such a high-content cluster of nuclei directly using one method, e.g. 3D watershed, does not provide results as good as those of the proposed procedure, which combines the modified 3D distance transform, 3D watershed and adaptive cells nuclei segmentation. In the end, it can be observed that all individual nuclei are separated in the specimen in Fig. 12a, regardless of the different degrees of connection between them (Fig. 12d).

### Adjustment of nuclear borders

**Fig. 13:**
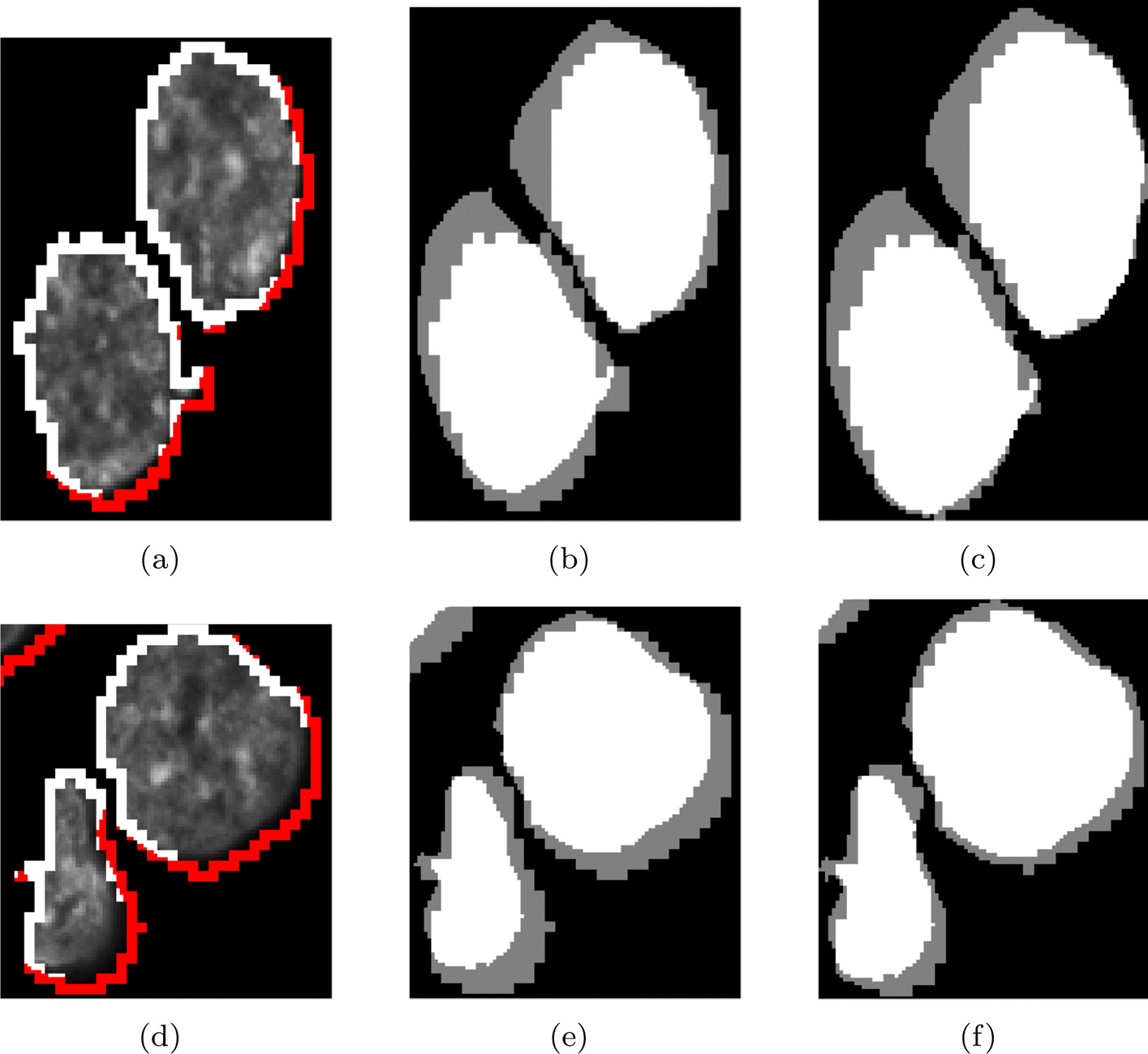
Example of final nucleus shape adjustment and surface voxel classification: **a**, **d** Voxels of the cell surface after classification at 1:1 scale (cell voxels—white; background voxels—red); **b**, **e** comparison with the expert’s mask without shape adjustment; **c**, **f** comparison with the expert’s mask after shape adjustment (white—expert’s mask)

The last stage of 3D image analysis comprises nucleus mask upsampling from 1:3 to 1:1 scale and nucleus surface refinement at 1:1 scale. Voxels inside cells are upsampled using the nearest neighbour interpolation. Initially, the surface voxels are upsampled in the same manner. However, since such upsampling yields a surface contour that is ${3}\times {3}\times {3}$ voxels thick, the contour thickness is reduced, and the nucleus shape is refined by surface voxel intensity thresholding. Voxels with intensity higher than $I_{Th} * NC_{MeanInt}$ (where $NC_{MeanInt}$ is the mean cell intensity excluding the surface, and $I_{Th}$ is the threshold) are merged with the cell body. Voxels that do not meet this criterion are assigned to the background. $I_{Th}$ was set experimentally using the training set: $I_{Th}$ values higher than 0.3 significantly reduced the nucleus volume. Lower $I_{Th}$ values had no effect on the final nucleus shape. Therefore, we set $I_{Th}$ at 0.3. By applying this approach, the mean JI increased on average by 6% in the test set, finally reaching 82% (Table 6). Figure 13 shows how this approach performed. As a result, the final shape and volume of the reconstructed cell more closely matches the expert’s mask (Fig. f13c–f), and the JI reaches higher values.

### Evaluation of results

In order to evaluate the segmentation accuracy, two assessment criteria previously used in the literature were applied [11, 41, 42]. The first criterion estimates the number of correct and incorrect cell detections [41]; the second estimates the accuracy of tracing [42]. Accuracy was assessed on the basis of 27 ground-truth images containing nuclei outlined by the expert (2367 in total). The expert’s masks generated in 2D layers were compared with the 3D binary masks of the segmented nuclei generated by the algorithm. The accuracy of the algorithms was assessed by comparing the masks in the layers selected by the expert.

**Fig. 14:**
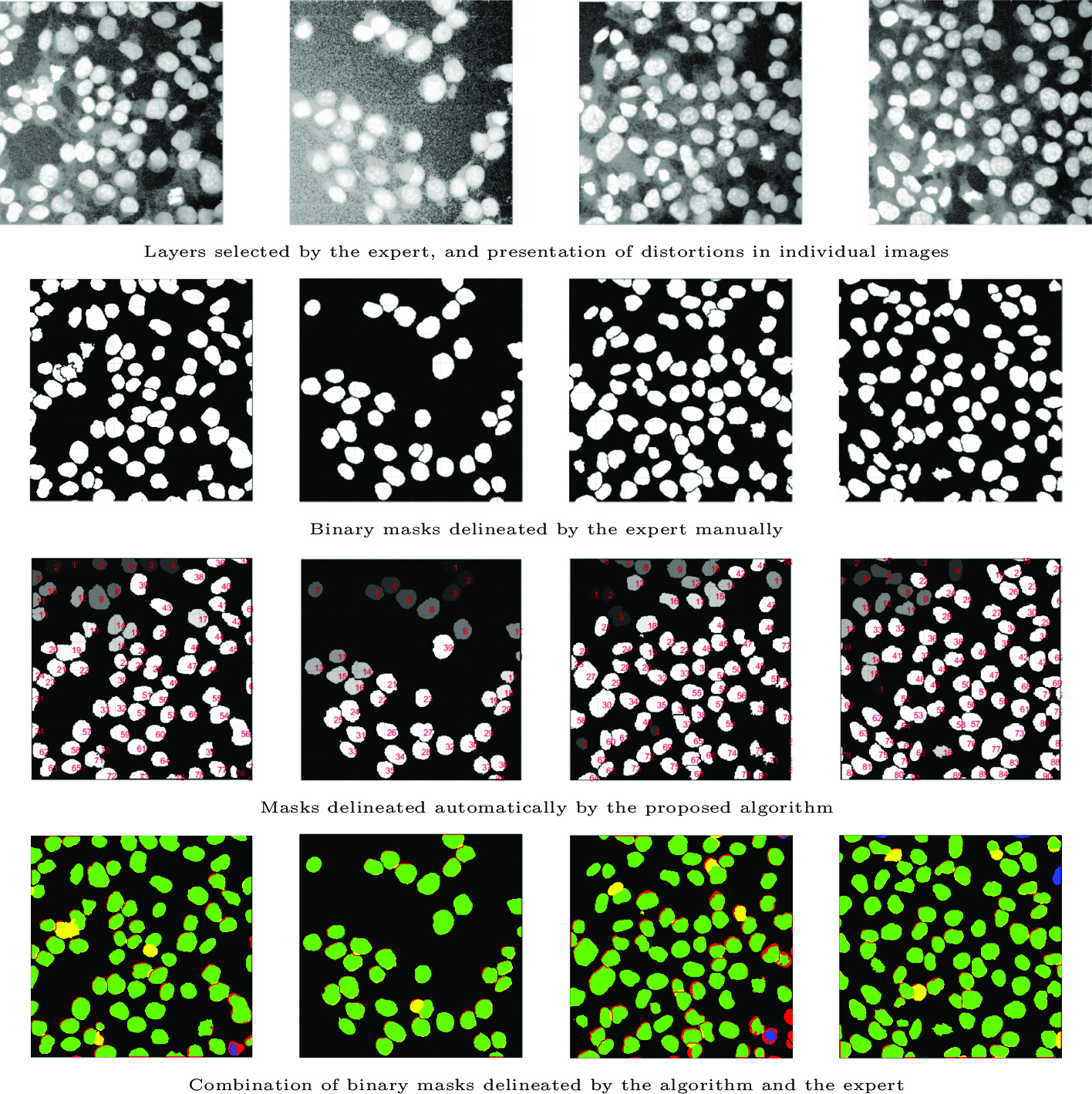
Example results of the analysis: examples from Fig. f2e, f, g, h. TP cases are marked in green; not properly separated nuclei are in yellow; FN cases are red; cases when the algorithm designated too small an area in relation to GT or FP cases are blue

The subsequent rows in Fig. 14 show the examined images; binary masks manually delineated by the expert; binary masks generated by the algorithm; and masks delineated by the algorithm and the expert combined in one image for each case.

**Fig. 15:**
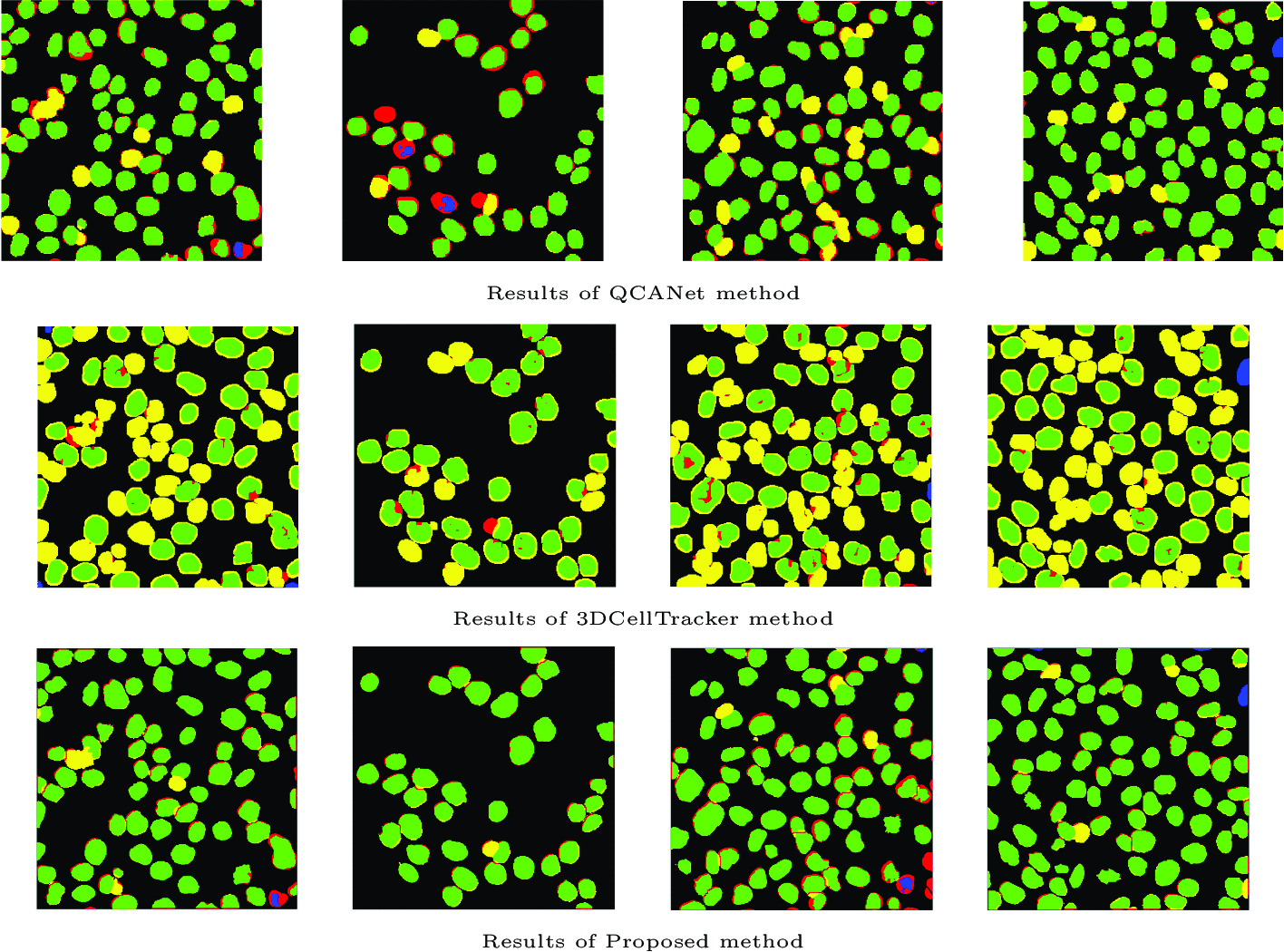
Comparison of segmentation results by reference methods and the proposed method. TP cases are marked in green; not properly separated nuclei are in yellow; FN cases are red; cases when the algorithm designated too small an area in relation to GT or FP cases are blue.

The algorithm, after a full 3D analysis of specimens, nuclei segmentation and reconstruction, compared the results for the layer selected by the expert; for this layer, it calculated the effectiveness, precision, specificity, F1-Score and JI. The above images (Fig. 14) show cellular specimens of varying confluency, high background noise, and the segmentation results obtained with the proposed algorithm. The cases in which the algorithm segmented the cell correctly are marked in green, whereas the cases in which the algorithm failed to effectively separate a group of cells or divided a single nucleus are highlighted in yellow. Red and blue are cases in which the mask delineated by the algorithm was too small or did not agree with the mask selected by the expert or was too small (less than 50% of the area of the expert’s mask).

It can be seen that in each case the background is completely eliminated, and the nuclei are properly separated from the background. There is a strong agreement between the generated binary masks of cells and the masks selected by the expert. The numbers of cell nuclei determined by the proposed method and the expert are almost identical. The algorithm rarely detects areas where cells are not actually present. In a few cases, it can be observed that the larger masks of cells delineated by the algorithm do not correctly cover the masks of nuclei traced by the expert (yellow in the bottom row). This is due to the fact that the algorithm did not properly separate the nuclei in the image. Therefore, when a large cell delineated by the algorithm covers more than 50% of a nucleus selected by the expert, it is not rechecked in terms of how much it covers another nucleus when calculating the effectiveness and agreement between the masks. *TP* refers to cases in which the algorithm correctly selected a nucleus mask which agreed (for over 50% of the area) with the mask selected by the expert. *FP* cases occur when the algorithm located an area that had been misclassified as a cell, or a single nucleus was unnecessarily divided. When comparing the algorithm results with the ground truth, if no nucleus matched the selected mask or the algorithm did not divide the nucleus group successfully, the cases were classified as *FN*. The JI for each nucleus was calculated as the quotient of the intersection and the sum of the binary masks of the cell segmented by the algorithm and marked by the expert. In contrast, the mean JI in the test image was calculated as the quotient of the sum of *JI* for all cells and the number of nuclei (Eq. 7).

7 $$\begin{aligned} JI_{Avg} = \frac{\sum _{i=1}^{c_{count}} \frac{A_i \cap B_i}{A_i \cup B_i} }{c_{count}} \end{aligned}$$

where:

- $JI_{Avg}$—average JI in the examined specimen
- $A_i$—binary mask of the i-th cell delineated by the algorithm
- $B_i$—binary mask of the i-th cell delineated by the expert
- $c_{count}$—number of detected nuclei

The results of the efficacy evaluation using the number of correct and incorrect detections as well as the JI are presented in Table 8 for the cases from Fig. 14.

**Table 8:** Results obtained for the images from Fig. 14

Image	TP	FP	FN	Prec	Sens	Alg.Num	GT.Num	F1-score	JI
Figure 14a	77	1	8	0.987	0.905	78	85	0.944	0.849
Figure 14b	39	0	1	1	0.975	39	40	0.987	0.856
Figure 14c	78	2	16	0.975	0.829	80	94	0.896	0.841
Figure 14d	88	3	6	0.967	0.936	91	94	0.951	0.847

The results obtained for all the images made it possible to compare the effectiveness of the proposed method with other reference solutions (described in the “Results” section—Tables 6, 7).

## Data Availability

Dataset and source codes are available at: https://doi.org/10.5281/zenodo.4462097.

## Abbreviations

HCS: High-content screening
$$NC_{S}$$: A single nucleus
$NC_{G}$: A group of nuclei
FN: False negative
FP: False positive
TN: True negative
TP: True positive
JI: Jaccard Index
